# Sleeping Rooms

**Published:** 1874-12

**Authors:** 


### SLEEPING ROOMS.

Observant readers know that serious and even fatal illness has resulted from persons having gone to sleep near an open window, from a sudden change of weather during the night. Persons living on "bottom lands," on river banks, and on lake and seashore, know very well that sickness is almost a certain result of sleeping with open windows. A writer, not a physician, states in a health journal, that when he takes cold, which he intimates occurs often, he wraps up his head, covers up in bed, and goes to sleep with the window fully hoisted. One thing is certain, that sleeping near an open window does not prevent him from taking cold. The writer's habit is to sleep with closed windows, and he has not had a "cold to speak of," or anything else, for a number of years.

Every intelligent reader should acquaint himself with a few general principles, for there are a few rules of universal application.

To sleep with the chamber windows open in miasmatic localities from May to November is to invite all miasmatic diseases, such as diarrhœa, dysentery, and various forms of autumnal fevers, from ague to "Yellow Jack." The reason is that the germs which originate these ailments begin to descend towards the earth at sundown, and begin to ascend in the morning, and there being a draught from outdoors through any open window into the room, these germs are blown in upon the sleeper, are breathed into the lungs, swallowed into the stomach, and find their way into the blood, making it, in some cases, almost as "black as tar" in a night, and so thick, that it will scarcely flow along the veins; this is congestive fever, a terror in the South only second to cholera.

If water freezes in a bed-room, or if it is even as cold as forty degrees, the carbonic acid generated in breathing becomes so condensed and heavy as to settle towards the floor, and is breathed by the sleeper, corrupting the blood with great rapidity. Persons become insensible in an hour or two, never wake up, and die before morning, from sleeping in a room in which is burning a pan of charcoal, for this generates the same deadly gas, only in a more concentrated form. This is the agent, also, which causes a sleeping apartment to "smell so close" on entering it in the morning. The colder a room is, the more dense, concentrated, and more deadly is the carbonic acid generated during sleep. Hence chamber windows should be closed in "bitter" cold weather; so, also, in all fever and ague localities and seasons for reasons given below, and on lake and seashore exposures, in consequence of the atmosphere being saturated with dampness; and no one will say that it is "healthy" to sleep in damp bed clothing indoors, however it may be on the open prairie or ship's deck. In their enthusiasm for fresh air, partially informed persons have often injured their health, having, however, for company, the great Benjamin Franklin, who brought on a fatal attack of sickness by insisting on sitting near an open window in his shirt-sleeves to get the pure air. It was this incident which led Thomas Jefferson to say, "Franklin was a fool."

A perfectly safe way to ventilate a sleeping apartment, especially for the young, the feeble, and the old, is to have the inner or chamber door open, and a lamp burning in the open fireplace; this creates a draught up the chimney, by the air coming in through the door and the crevices about the windows, seeking the vacuum which the flame makes, and carrying before it into the fire-place and up the chimney the heaviest and most contaminated air. Hence, in very cold weather, it is "healthier" to have a little fire in a sleeping room; and during the winter, every person who can afford it will be the better for getting up and dressing by a good blazing fire. The comfort of it any one must acknowledge, and health and life are promoted and prolonged by what are called comforts; the comforts of good living, having plenty of the best food, prepared in the best manner. No man is benefited by exposures or hardships, for their tendency in all cases is to engender disease and shorten life.

In all furnace or stove-heated houses, the chamber door should be closed before bed-time, to keep out the furnace heat, and a window should be opened an inch or two, so as to gradually cool the room, without making it freezing cold, at the same time admitting enough fresh and pure air to supply what has been used up by the sleeper, and also to carry with it on its passage to the fire-place and up the chimney all contaminating materials. The general rule is to keep the chamber windows closed in cold weather, during epidemics in lake and seashore localities, and wherever spring and fall diseases are prevalent.

